# The 2026 Andes virus outbreak: What genomic stability reveals about virulence

**DOI:** 10.1080/21505594.2026.2698897

**Published:** 2026-07-09

**Authors:** Justin L. Kung, David Jesse Sanchez

**Affiliations:** Department of Molecular, Cellular and Developmental Biology, Yale College, New Haven, CT, USA; Pharmaceutical Sciences Department, Western University of Health Sciences, Pomona, CA, USA

**Keywords:** Andes virus, hantavirus cardiopulmonary syndrome, genomic stability, viral virulence, zoonotic spillover, outbreak surveillance

When a new outbreak of hantavirus, a high-mortality pathogen, appeared on an international cruise ship, the reflex was to ask if a new genetic variant has emerged. The MV Hondius Andes hantavirus outbreak of May 2026 presents a different lesson, as it is an outbreak of a genetically stable virus. Andes hantavirus (ANDV) remains one of the most lethal zoonotic illnesses in the Americas. ANDV is an etiologic agent of hantavirus cardiopulmonary syndrome (HCPS), with case-fatality rates ranging between 25% and 40%, although some specific cases have been reported with fatality rates over 50% [1,2]. As of 28 May 2026, the World Health Organization update reports 13 cases of ANDV, with 11 confirmed and 2 probable cases, and 3 deaths. This report also notes that all cases to date were passengers or crew members, with the global public risk remaining low.

ANDV is also capable of sustained person-to-person transmission, first documented in Argentina in 1996 and observed during the 2018–2019 outbreak in Epuyén, a rural community in the Chubut Province of southwestern Argentina [2]. Following a spillover event from the rodent reservoir *Oligoryzomys longicaudatus*, three symptomatic individuals attending crowded social events drove transmission, causing 34 confirmed infections and 11 deaths, with a median reproductive number (R_0_) of 2.1. ANDV person-to-person spread occurs primarily during or shortly after the end of the prodromal phase and requires close and prolonged contact, as exemplified by a confirmed secondary case arising from a 14-hour shared bus ride in 2002 [3]. In this instance, Martinez et al. concluded that the route of transmission was via small-particle infectious saliva or respiratory aerosols during close contact. Moreover, the most probable period of viral spread is in the days before an infected individual seeks medical attention, placing household and social contacts at greater risk than treating clinicians. Specifically, close household contacts who share a bed or bedroom with an index case-patient face a 10-fold higher risk of infection than other household contacts [4].

The strain responsible for the 2018–2019 outbreak is closely related to the 2026 ANDV strain (ANDV/Switzerland/Hu-3337/2026) [5] (see Figure 1). The 2026 strain genomes are strikingly similar across cases, with identical S and M segments and only two synonymous SNPs in the L segment, consistent with past ANDV isolates. The outbreak itself is consistent with a single zoonotic spillover followed by limited human-to-human transmission in the closed-contact setting of maritime travel. The most important genetic fact regarding the 2026 outbreak is not novelty, but continuity. That is important because it forces us to reconsider how we think about Andes virus virulence and what genetic stability actually indicates epidemiological risk.

**Figure 1.**
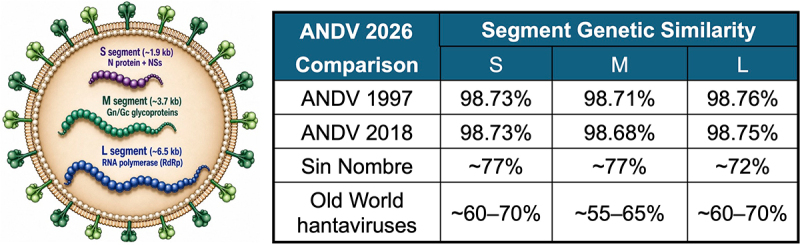
Genomic architecture and evolutionary conservation of Andes virus (ANDV) 2026.

## Andes tropism as a key virulence factor

For the 2026 outbreak of ANDV, much of the initial focus was on mutations from earlier Argentine outbreaks thought to confer transmission advantage: NSs Q40R, NSs N47S, and a glycoprotein change at T641I. Public Health Ontario’s genomic review notes the conspicuous absence of all three in the 2026 sequences. The Swiss genome also places the 2026 virus in clade 3 rather than clade 2, which was linked to the 2018–2019 outbreak. This is a key point since this 2026 virus does not carry previously identified mutations associated with person-to-person transmission.

The fundamental division in hantavirus pathogenesis is geographic, with Old World hantaviruses endemic in Asia associated with hemorrhagic fever with renal syndrome, while New World hantaviruses like ANDV in the Americas are associated with cardiopulmonary syndrome centered on pulmonary capillary leak. A 2025 comparative study in human cell culture revealed differences in tropism with ANDV, showing broad infectivity across the pulmonary endothelium, lung epithelium, cardiomyocytes, and astrocytes, while the related New World virus Sin Nombre replicated mainly in the pulmonary endothelium [6]. In contrast, the Old World Hantaan virus is concentrated in kidney cells. These differences in tissue tropism align with the distinct clinical syndromes where New World viruses are associated with pulmonary disease and Old World viruses are associated with renal disease. As with all other viruses, the first layer of virulence is based on which tissues the virus can productively infect.

ANDV and Sin Nombre virus both use cellular protocadherin-1 (PCDH1) as their entry receptor [7,8]. This protein is expressed in airway and vascular endothelium, aligning with the targets for cardiopulmonary disease manifestation [4]. CRISPR-based receptor depletion experiments found that PCDH1 loss strongly impairs New World, but not Old World, hantavirus entry into endothelial cells. This supports why ANDV causes pulmonary disease, while the Old World Hantaan hantavirus concentrates in renal tissue without significant disease of the lungs. This tight link between receptor specificity and disease makes virulence difficult to alter with small genetic changes.

## Andes glycoproteins and immune evasion

Receptor usage alone does not explain ANDV virulence; the 2026 Swiss sequence carries multiple amino acid changes, including V8A, I114V, H294Y, and others, along with NSs mutations such as Q5R and E33G [5]. Yet none has been proven necessary or sufficient for enhanced human spread. A preliminary analysis found no strong signal of human-associated adaptation.

Where ANDV becomes distinct is immune evasion. The nucleocapsid protein carries an innate immunity regulator with interferon-regulatory activity at the C-terminus of the N protein [9]. ANDV NSs antagonize MAVS-dependent type I interferon induction, which may facilitate early viral replication and contribute to the development of viremia [10]. Whether this activity directly contributes to respiratory shedding and person-to-person transmission remains unresolved. Recent evidence shows ANDV RNA in saliva and respiratory secretions during early symptomatic infection, overlapping peak transmission windows [11,12]. One hypothesis is that suppression of interferon responses permits greater early viral replication and viremia, increasing the likelihood of viral shedding during the prodromal and early symptomatic phases when infected individuals remain ambulatory and capable of transmitting virus.

## Differences in New World hantaviruses

Comparing the two major New World hantaviruses associated with human disease, ANDV to Sin Nombre infection from 1993, shows what is shared and what is not. Both use PCDH as a receptor and are linked to pulmonary syndrome. Their genomes shared > 85% amino acid identity in L and S segments, indicating close lineages [5,13]. Yet infection of Sin Nombre in hamsters is nonpathogenic with no detectable viremia, while ANDV is lethal [13]. In human organoid systems, ANDV shows broader tropism. This 2026 outbreak of ANDV demonstrates this same phenotype with no new mutations, no sudden shift, just intrinsic properties in an unusual maritime setting.

Here virulence is a systems property, not a single substitution changing receptor usage, tropism, immune antagonism, viremia dynamics, and transmission potential. Also, the 2026 ANDV genomes are stable with no newly acquired transmission mutation present. Instead, the virus carries accumulated tropism and immune-evasion capacities that define New World hantavirus biology. In a closed maritime environment with sustained contact, occasional human-to-human transmission becomes consistent with intrinsic properties, not recent evolutionary leaps. This stability in a way is reassuring as it means ANDV behavior is predictable and containment strategies grounded in our understanding of the virus will be effective.

## Proven containment and outbreak control of ANDV

During Epuyén, Argentine public health officials enforced isolation of symptomatic individuals and self-quarantine for high-risk contacts. These measures were remarkably effective: the median reproductive number was reduced from 2.1 to 0.96, suppressing transmission and enabling early detection of HCPS. A 2011 nosocomial outbreak in southern Chile demonstrated that monitoring all close contacts, including healthcare personnel who directly or indirectly interact with infected patients, is essential to prevent healthcare-associated transmission. Such measures were proven necessary to curb further outbreaks [4].

Surveillance strategy requires integration of clinical and laboratory approaches. While serologic testing (IgM) has been traditionally relied upon, it should not be used alone; a negative test may reflect low early viral titers [14]. RT-PCR on blood cells is most useful in the first 7–10 days of illness and can detect infection before or when IgM appears, especially in the first 72 hours when antibodies may still be negative [14]. This combined approach with early molecular detection and serology as confirmation can maximize case identification during the critical window when isolation and treatment are most effective.

Droplet precautions, including the use of N95 respirator masks, are recommended to prevent further transmission, particularly in healthcare settings where prolonged exposure to patients’ respiratory secretions is unavoidable [2,4]. Clinical decisions should prioritize exposure history, early RT-PCR diagnosis, rapid isolation of symptomatic individuals, and methodical contact monitoring rather than hunting novel “transmission mutations” as sequence surveillance showing the 2026 outbreak clusters as a single lineage.

ANDV did not suddenly become more transmissible, and the outbreak setting was unusual. The 2026 outbreak is not a cautionary tale of emergence, but a reminder that scientifically grounded surveillance and proven public health practices remain our most reliable tools to stop outbreaks. ANDV transmission control depends on recognizing the inherent human-to-human transmission ability of ANDV, and implementing isolation, contact tracing, and diagnostic protocols. The stability of the 2026 genomes and predictability of transmission in this maritime setting underscores how understanding virus biology informs epidemiological response.

The ANDV genome consists of three RNA segments, S (~1.9 kb), M (~3.7 kb), and L (~6.5 kb), encoding the nucleocapsid (N) and NSs proteins, Gn/Gc glycoproteins, and RNA-dependent RNA polymerase (RdRp), respectively. Comparison of the genomic similarity of the 2026 outbreak strain to the historical ANDV isolates shows greater than 98% identity across all three segments [5]. This indicates minimal genetic divergence despite nearly three decades of circulation. The virion in the figure was created with assistance from ChatGPT using OpenAI (GPT-5 series, accessed 2026) for conceptual development and visualization refinement of virion. The final figure was subsequently modified and finalized by the author.

## Data Availability

Data sharing is not applicable to this article as no new data were created or analyzed in this study.
